# Advancing the science of recruitment for Alzheimer’s clinical trials: Challenges and opportunities

**DOI:** 10.1016/j.tjpad.2025.100230

**Published:** 2025-06-12

**Authors:** Paul Aisen, Desi Peneva, Maria-Alice Manetas, Mireille Jacobson, Dana Goldman, Niranjan Bose, Phyllis Barkman Ferrell, VK Vu, Rema Raman

**Affiliations:** a Alzheimer’s Therapeutic Research Institute, Keck School of Medicine, University of Southern California, San Diego, CA, USA; b Leonard D. Schaeffer Center for Health Policy and Economics, University of Southern California, Los Angeles, CA, USA; c Price School of Public Policy, University of Southern California, Los Angeles, CA, USA; d Leonard Davis School of Gerontology, University of Southern California, Los Angeles, CA, USA; e Mann School of Pharmacy and Pharmaceutical Sciences, University of Southern California, Los Angeles, CA, USA; f Gates Ventures LLC, Kirkland, WA, USA

**Keywords:** Alzheimer’s disease, Clinical trials, Inclusive recruitment, Retention

## Abstract

Despite recent advancements in Alzheimer’s disease therapeutics and diagnostics, significant challenges remain in accelerating participant recruitment—particularly among diverse populations—and ensuring equitable access to clinical trials. This paper summarizes discussions and recommendations from the 2024 Roundtable on *Advancing the Science of Recruitment for Inclusive Alzheimer’s Disease Clinical Trials*, hosted by the USC Clinical Trial Recruitment Lab (CTRL). Bringing together 40 leading experts from across the Alzheimer’s clinical trial ecosystem in the U.S., including thought leaders from academia, industry, government and philanthropy, the Roundtable examined critical barriers to inclusivity and explored emerging recruitment approaches. Discussions highlighted the need for strategies that expand patient access, remove systemic barriers, and foster inclusivity in clinical research.

## Highlights

• Highlights urgent need to improve Alzheimer’s clinical trial recruitment and inclusivity.
• Examines barriers limiting representative participation in Alzheimer’s clinical research.
• Summarizes insights from 2024 USC Roundtable of 40 cross-sector Alzheimer’s experts.
• Presents emerging strategies to boost inclusive recruitment and participant access.
• Recommends actions to remove systemic obstacles and improve trial representation.

## 1 Introduction

Recent advancements in pharmacology and biomarker research have significantly reshaped the landscape of Alzheimer’s clinical research, creating both new opportunities and challenges in clinical trial recruitment. The approval of second-generation anti-amyloid therapies such as lecanemab and donanemab has demonstrated the potential for disease modification, slowing clinical progression by approximately 30 % in early-stage patients [1,2]. Furthermore, the emergence of blood-based biomarkers—particularly plasma pTau217—offers a more accessible and cost-effective alternative to traditional amyloid PET or cerebrospinal fluid (CSF) tests, enhancing the feasibility of early detection and trial enrollment [3].

Despite these scientific advancements, Alzheimer’s clinical trials continue to face significant recruitment challenges that hinder research progress [4]. As of 2024, an estimated 51,000 participants are needed to fully populate the 164 Alzheimer’s clinical trials currently in the pipeline [5]. However, 2020 estimates indicate that out of approximately 90 million Americans potentially eligible for preclinical, prodromal, or mild Alzheimer’s clinical trials, only about 11,000 successfully enroll in clinical studies [6]. As a result, Alzheimer’s clinical trials are slow to enroll sufficient sample size [7], creating a critical barrier to expeditiously translating scientific discoveries into effective treatments. Furthermore, Alzheimer’s clinical trials struggle to recruit diverse study participants. Although people from underrepresented groups face a higher risk of Alzheimer’s disease and related dementias, they are less likely to participate in clinical research [8]. The urgency of improving clinical trial recruitment is magnified by the rising prevalence of Alzheimer’s dementia, which currently affects around 7 million Americans—a number projected to nearly double to 13 million by 2050 [9].

To address the complex barriers to participation in Alzheimer’s clinical trials, the USC Schaeffer Institute and the Alzheimer’s Therapeutic Research Institute (ATRI) at the Keck School of Medicine established the Clinical Trial Recruitment Lab (CTRL). CTRL generates evidence to inform and enhance clinical trial recruitment, retention, and representation. This is done in part by coordinating resources and expertise to conduct pilots that test effective recruitment and retention strategies. In addition, CTRL actively disseminates and shares findings and recruitment best practices. CTRL fosters collaboration across the field to break down silos, build networks, and share recruitment strategies that advance inclusive and timely Alzheimer’s clinical trials. As part of the American Heart Association’s Strategically Focused Research Network on the Science of Diversity in Clinical Trials, CTRL also houses the Alzheimer's Trial Recruitment Innovation Lab (ATRIL), which aims to accelerate the enrollment of a more representative population in preclinical and prodromal Alzheimer’s clinical trials through an evidence-based science, community engagement, and training.

In November 2024, CTRL hosted a two-day Roundtable on *Advancing the Science of Recruitment for Inclusive Alzheimer’s Disease Clinical Trials*. The Roundtable convened 40 leading experts across the Alzheimer’s clinical trial ecosystem in the U.S., including thought leaders from academia, industry, government and philanthropy, to address critical challenges and explore emerging recruitment strategies to advance inclusive trial recruitment nationwide. The discussions highlighted the need for effective approaches that enhance patient access, remove systemic barriers, and foster inclusivity in clinical research. While the Roundtable focused on inclusive recruitment, many of the identified barriers and proposed strategies—such as innovations in screening, infrastructure development, and workforce expansion—are broadly applicable and critical to improving overall recruitment and retention in Alzheimer’s clinical trials. Strengthening recruitment systems and reducing structural barriers not only enhance trial efficiency but also has the potential to create more equitable access for underrepresented populations. This paper summarizes the key barriers to inclusive recruitment, emerging recruitment strategies, and policy recommendations and considerations that came out of the Roundtable.

## 2 Key barriers and challenges

Roundtable participants engaged in a discussion about the complex, interconnected barriers and challenges hindering inclusive recruitment and retention in Alzheimer’s disease clinical trials.

### 2.1 Historical trust barriers

Historical trauma and systemic inequities have fostered a deep-seated distrust of medical research, particularly among racial and ethnic minority communities. Past exploitation and unethical research practices have left lasting scars, influencing participation rates today. Roundtable participants emphasized that this historical context must be thoughtfully acknowledged and integrated into recruitment strategies to rebuild trust and actively promote more inclusive participation in clinical trials.

### 2.2 Accessibility barriers

Geographic barriers, transportation challenges, work and scheduling conflicts, and financial constraints all significantly impact participation in clinical trials. For many individuals, especially in rural or underserved areas, the distance to trial sites makes participation difficult. Those without reliable or affordable transportation options, or access to public transit, face additional hurdles. Many potential participants also have rigid work schedules and cannot afford to take time off, which makes attending study visits a challenge. Beyond lost wages, there are additional costs for childcare, transportation, and other incidental expenses that participants may struggle to cover, which further limits their ability to engage in clinical trials.

### 2.3 Institutional and structural barriers

Research centers and sites encounter significant institutional challenges that hinder recruitment efforts. Many sites struggle with securing sustainable funding for community engagement between trials, resulting in fragmented and inconsistent relationships with local communities. The current funding model, which allocates resources solely for specific trials rather than continuous community engagement, exacerbates gaps in building relationships. Complex trial protocols and rigid eligibility criteria also often exclude individuals with comorbidities, which are more prevalent in underserved populations [10]. Moreover, multiple layers of screening can lead to substantial participant drop-off between initial interest and pre-screening completion.

### 2.4 Cultural and linguistic challenges

A persistent shortage of culturally appropriate recruitment materials and assessment tools validated for diverse populations further contributes to representation challenges. Many trials lack materials in languages other than English, and when translations are available, they may not be culturally nuanced or appropriate. Cognitive assessment tools often include cultural references that may be unfamiliar to certain populations, which can impact test performance and eligibility determinations.

### 2.5 Infrastructure and staffing issues

The concentration of Alzheimer’s clinical trials in urban medical centers creates significant accessibility barriers for patients who receive care in community settings [11]. Additionally, staff retention challenges, particularly among clinical coordinators and research personnel, disrupt the continuity essential for effective community engagement. High turnover rates hinder relationship-building with local populations and introduce inefficiencies into the recruitment process.

### 2.6 Diagnostic and biomarker disparities

The field faces distinct challenges related to biomarker testing and diagnosis. Recent data indicate lower amyloid positivity rates among certain racial and ethnic groups, raising concerns about the applicability of current diagnostic criteria and the potential variability of disease manifestation across populations [12]. Furthermore, the presence of co-pathologies, such as vascular dementia, adds complexity to diagnosis and complicates eligibility determinations for clinical trials.

## 3 Emerging recruitment strategies

Roundtable participants discussed emerging recruitment approaches for improving inclusion in Alzheimer’s clinical trials. Additionally, CTRL pilot teams shared their research goals, key insights, and lessons learned.

### 3.1 Innovation in screening and detection

Advancements in screening and detection technologies have the potential to transform the landscape of Alzheimer’s clinical trials, making trial recruitment and diagnostics more accessible and efficient. The development of blood-based biomarkers marks a significant advancement in enhancing the accessibility and cost-effectiveness of screening. In the AHEAD Study, for example, plasma testing incorporating pTau217 was used as a pre-screening tool to identify patients with elevated amyloid pathology, which allowed for more efficient and accurate selection for confirmatory PET imaging [13]. This approach demonstrated the potential to reduce screen failure rates from 75 % to below 25 % [14]. Integrating blood-based biomarkers into clinical trials could significantly improve efficiency, reduce costs, and alleviate the burden on both clinical trial sites and participants. Further, advancements in digital patient engagement also play a role in improving detection, communication, coordination, and overall participant experience. Remote cognitive assessments have streamlined the screening process, minimizing participant burden. Studies have demonstrated that unsupervised digital memory assessments can be effectively used for diagnosis and prognosis in Alzheimer's disease, potentially in combination with plasma biomarkers [15]. In another example, one CTRL pilot is assessing digital detection tools to identify potential participants through electronic health records, achieving an 80 % accuracy rate in detecting undiagnosed dementia or mild cognitive impairment [16]. These passive digital markers can screen 5000 patient records in under two hours—a significant improvement over the 17 days required for traditional brief assessments. Identified individuals are then contacted through a secure, patient-centered messaging system designed to encourage follow-up cognitive assessments, and with consent, offer clinical trial opportunities. This approach facilitates both medical evaluation and research recruitment within primary care settings. While these technological solutions are gaining momentum, their impact on Alzheimer’s clinical trials, particularly in diverse and underrepresented populations, is still being evaluated. As these innovations continue to evolve, their integration into clinical trials holds great promise for accelerating diagnosis, enhancing recruitment, and ultimately advancing Alzheimer’s disease research.

### 3.2 Community-based approaches

Community-based recruitment strategies have proven to be effective in increasing clinical trial participation, particularly among historically underrepresented populations. Several CTRL pilots are evaluating community engagement and recruitment models to better understand and enhance their effectiveness. One such pilot explores the use of group screening in community settings, particularly through partnerships with faith-based organizations, to expand access and foster engagement. Research has demonstrated that these community-driven recruitment approaches effectively improve trial participation among racial and ethnic minority groups [17]. By integrating educational outreach with immediate screening opportunities, these initiatives help lower participation barriers and shorten the gap between initial interest and enrollment. Embedding these strategies into clinical trial infrastructure enables research teams to establish sustainable, community-centered recruitment models that drive long-term engagement and inclusivity.

Creating a clinical trial infrastructure within existing clinical care and community health settings can strengthen trust, improve accessibility, and support more sustained engagement over time. This approach would move beyond episodic interactions and instead fosters continuity of care by aligning research with the routine delivery of care. Such integration could leverage the insights from CTRL pilots to reduce participation barriers, enhance equity, and establish long-term partnerships between research institutions and the communities they aim to serve. Understanding how best to operationalize this integration across diverse settings is a critical area for ongoing exploration.

Decentralized trial sites have significantly improved access to clinical research for individuals in remote areas. By leveraging telemedicine and mobile technologies, decentralized clinical trials allow many patients to participate from the comfort of their homes or local healthcare facilities, enabling more seamless data collection and remote monitoring. However, it is important to recognize that this model depends on reliable access to internet service and digital devices—resources that are not equally accessible to all, especially in rural communities. Lastly, mobile screening units have effectively brought testing capabilities directly to community settings, helping to overcome both technological and transportation barriers and further enhancing inclusivity in clinical research.

### 3.3 Financial and logistical considerations

Financial incentives for clinical trial participation were a topic of debate. While FDA guidance acknowledges that compensation is a common practice, it cautions against the risk of “undue influence” [18,19]. Empirical research on the role of different levels and types of financial incentives is, however, still nascent. One CTRL pilot addressing this issue found that small, guaranteed payments (e.g., $25) led to modest increases in enrollment rates, whereas larger, lottery-style incentives ($2500) were less effective [20]. Beyond financial incentives, roundtable participants suggested that recruitment strategies may need to consider a multi-level approach to participant support—including providing direct services related to transportation, childcare, and meals—while considering remuneration that is tailored to address individual barriers without compromising ethical standards. More specifically, programs that provide transportation assistance through pre-arranged services, rather than post-trip reimbursement, may be more effective in removing financial barriers. Clinical sites providing childcare support during trial visits have seen a notable increase in participation from caregivers of young children. Flexible scheduling, including evening and weekend appointments, has made participation more feasible for working individuals. Programs that offer compensation for caregivers and study partners recognize the broader impact of trial participation on families. Differential financial incentives and assistance that consider varying levels of burden across diverse populations may help address socioeconomic barriers to participation. Roundtable participants generally agreed that further research is needed to assess not only the impact of financial remuneration but also the role of non-monetary barriers. For example, offering remote visits and eliminating unnecessary procedures can reduce participant burden, making this a promising strategy to enhance inclusive recruitment.

### 3.4 Cultural and linguistic adaptations

An increasing number of trials are adopting culturally sensitive approaches to enhance recruitment and retention among underrepresented populations. The development of culturally tailored recruitment materials has improved communication with diverse communities and has been shown to significantly increase interest in clinical trial participation. National surveys revealed that culturally tailored outreach materials resulted in a median 12.5 % increase in individuals expressing strong interest in Alzheimer’s clinical trials across Black/African American, Hispanic/Latino, and Asian American and Pacific Islander communities [21]. One CTRL pilot responds to the rising prevalence of Alzheimer’s disease in American Indian and Alaska Native (AI/AN) populations by developing and testing culturally tailored recruitment materials. While efforts to engage AI/AN populations in Alzheimer’s clinical trials remain limited, this pilot represents an important step forward. The pilot seeks to establish a culturally relevant theoretical framework that will inform future outreach efforts, ultimately enhancing AI/AN participation in Alzheimer’s clinical trials. Further, integrating native speakers as clinical raters and implementing culturally tailored educational programs can improve assessment accuracy and boost enrollment among non-English-speaking participants [22]. Collectively, these initiatives underscore the importance of cultural and linguistic adaptations in Alzheimer’s clinical trials to foster inclusivity and improve health outcomes across diverse populations.

## 4 Policy recommendations and considerations

### 4.1 Sustainable funding mechanisms

Policy changes should prioritize sustainable funding structures that support both community engagement and trial infrastructure. Social capital, like community trust, is challenging to re-establish before each Alzheimer’s clinical trial. Establishing permanent community-based research networks, based on the successful models used in oncology and infectious disease, could provide long-term platforms for consistent recruitment [23]. Securing dedicated funding streams for community engagement specialists, independent of specific trials, would foster continuous, trust-based relationships with local communities. Furthermore, allocating direct funding to community organizations as equal partners—rather than as sub-awardees—could strengthen collaborative partnerships and ensure greater community buy-in. Additionally, investing in staff retention and professional development is crucial for preserving institutional knowledge and nurturing community relationships.

### 4.2 Regulatory framework enhancements

Regulatory policies should be strengthened to ensure meaningful representation in trial participation. Some suggested that the implementation of actionable diversity plans with clear, measurable outcomes would create specific benchmarks for success. Developing standardized metrics for tracking and reporting representation in trial participation would enable more consistent comparisons across studies and institutions. Introducing accountability measures for trials that fail to meet diversity goals would underscore the importance of inclusive recruitment. These measures, however, also risk introducing added complexity and cost into trial design and reporting. Others suggested that establishing guidelines for equitable compensation and robust support for trial participants would help standardize best practices across studies.

### 4.3 Infrastructure development

Investing in community-based research networks is essential for improving Alzheimer's trial recruitment. Some suggested that establishing permanent trial networks can help overcome socioeconomic barriers through decentralized trials, affordable screening tools, and expanded trial sites in underrepresented communities. Sustainable funding and strong community engagement will further enhance recruitment efforts. For instance, policies could encourage the integration of clinical trials into community and primary care settings by supporting the development of community-based research networks. Embedding clinical trials within primary care can increase participation, while training healthcare providers in trial protocols will expand the number of qualified recruitment sites. Additionally, investing in technology infrastructure, translation services, and culturally adapted outreach strategies will improve accessibility for diverse populations. Successful models from the National Cancer Institute (NCI) and the U.S. Department of Veterans Affairs (VA) demonstrate the value of building lasting community-based research networks, including sustained partnerships with community organizations as equal partners in trial design and execution. For example, the VA has built effective networks that engage diverse populations, while the NCI’s Community Oncology Research Program (NCORP) supports the integration of cancer prevention and treatment research into underserved communities [23,24]. These networks have played an important role in recruiting and retaining underrepresented populations in clinical trials, providing valuable insights for developing sustainable, community-driven models that can be adapted in Alzheimer’s research.

### 4.4 Workforce development

Some suggested policy initiatives to develop a diverse and well-trained clinical trial workforce through holistic approaches. Supporting programs that train diverse clinical trial professionals helps cultivate a workforce that reflects the populations in future trials. Providing cultural competency training equips staff to engage effectively with diverse communities. Establishing resources for staff wellness and retention programs fosters long-term relationships with communities. Additionally, creating incentives for building community-based research expertise will drive innovation in recruitment and retention strategies.

## 5 Limitations

This manuscript summarizes the key themes and recommendations that emerged from the Roundtable discussions, reflecting the perspectives of participating stakeholders on barriers and strategies to enhance clinical trial participation. It highlights broad priorities and emerging ideas rather than providing an exhaustive or detailed set of implementation guidelines. Ongoing work is needed to develop more specific, actionable approaches to translating these insights into practice. Future efforts should focus on not just expanding these discussions but also producing targeted guidance to support researchers, sponsors, and policymakers in advancing equitable and inclusive clinical research.

## 6 Conclusion

Improving inclusivity in Alzheimer's clinical trials requires a multifaceted approach that tackles both structural barriers and infrastructure challenges. Achieving success will depend on sustained investment in community relationships, innovative recruitment strategies, and supportive policy initiatives. As new therapeutics and diagnostics continue to emerge, ensuring equitable access to clinical trials is critical to addressing disparities in Alzheimer's disease treatment and outcomes.

Looking ahead, the focus should shift to implementing evidence-based recruitment strategies while maintaining the flexibility to adapt to local community needs. This will require ongoing collaboration among sponsors, researchers, healthcare providers, community organizations, and policymakers to build sustainable and inclusive clinical trial infrastructure for Alzheimer’s disease.

## Funding

This work was supported by Gates Ventures; the American Heart Association [grant numbers 946223, 964386]; and the USC Leonard D. Schaeffer Institute for Public Policy & Government Service.

## Declaration of generative AI and AI-assisted technologies in the writing process

During the preparation of this work the authors used OpenAI’s ChatGPT in order to improve the readability of the manuscript. After using this tool, the authors reviewed and edited the content as needed and take full responsibility for the content of the published article.

## CRediT authorship contribution statement

**Paul Aisen:** Writing – review & editing, Supervision, Funding acquisition, Conceptualization. **Desi Peneva:** Writing – review & editing, Writing – original draft, Supervision, Project administration, Funding acquisition, Conceptualization. **Maria-Alice Manetas:** Writing – review & editing, Writing – original draft, Project administration, Conceptualization. **Mireille Jacobson:** Writing – review & editing, Supervision, Project administration, Funding acquisition, Conceptualization. **Dana Goldman:** Writing – review & editing, Supervision, Funding acquisition, Conceptualization. **Niranjan Bose:** Writing – review & editing, Supervision, Resources, Conceptualization. **Phyllis Barkman Ferrell:** Writing – review & editing, Supervision, Project administration, Conceptualization. **VK Vu:** Writing – review & editing, Supervision, Project administration. **Rema Raman:** Writing – review & editing, Supervision, Funding acquisition, Conceptualization.

## Declaration of competing interest

The authors declare the following financial interests/personal relationships which may be considered as potential competing interests:

Paul Aisen reports grants from National Institute of Health, Lilly, Alzheimer's Association; consulting for Merck, Roche, Genentech, Abbvie, Biogen, Checkpoint, Immunobrain, Arrowhead; and research collaboration with Eisai and CogRX. Desi Peneva and Maria-Alice Manetas report grants from American Heart Association and Gates Ventures. Mireille Jacobson reports grants from American Heart Association, Gates Ventures; personal fees from expert testimony for various private clients. Dana Goldman reports grants from American Heart Association, Gates Ventures, Alexion, Amgen, Biomarin, Blue Cross Blue Shield of Arizona, Blue Cross Blue Shield of Massachusetts, BrightFocus, Bristol Myers Squibb, California Hospital Association, Cedars-Sinai Health System, Charles Koch Foundation, CommonSpirit, Edwards Lifesciences, Genentech, Gilead Sciences, Incyte, Johnson & Johnson, Lilly, National Institute on Aging, National Institute of Diabetes and Digestive and Kidney Diseases, Novartis, Pfizer, RA Capital, Roche; personal fees from Edwards Lifesciences, National Railway Labor Conference, GRAIL; equity in EntityRisk. Phyllis Barkman Ferrell is a retiree and minority shareholder of Eli Lilly & Company and reports personal fees from Davos Alzheimer’s Collaborative, Gates Ventures, Morningside, Alzheimer’s Drug Discovery Foundation, SiteRx, StartUp Health, Global Alzheimer’s Platform Foundation. Rema Raman reports grants from American Heart Association, Gates Ventures, Eisai, Alzheimer's Association. Niranjan Bose and VK Vu report no conflict of interest.
